# A Diagnostic Dilemma: What Not to Miss in Methaemoglobinaemia

**DOI:** 10.7759/cureus.96966

**Published:** 2025-11-16

**Authors:** Muhammad M Isar, Muhammad Hassan, Areesha Javed, Monowara Rahman Shipi, Harshvardhan P Thakar

**Affiliations:** 1 Acute Medicine, Pilgrim Hospital, United Lincolnshire Teaching Hospitals NHS Trust, Boston, GBR; 2 Gastroenterology, Pilgrim Hospital, United Lincolnshire Teaching Hospitals NHS Trust, Boston, GBR

**Keywords:** dapson, methaemoglobinaemia, oncology, pneumocystis jiroveci pneumonia prophylaxis, tissue hypoxia

## Abstract

Methaemoglobinaemia is a rare disorder in which haemoglobin loses its ability to deliver oxygen to tissues due to oxidation of haem iron. It may be congenital or acquired, most commonly secondary to medication. Patients present with apparent hypoxia unresponsive to oxygen therapy, which can be life-threatening at higher levels. Here we describe a case of dapsone-induced methaemoglobinaemia, which was initially overlooked due to possible sinister pathologies, leading to extensive investigations and repeated admissions, underscoring the importance of careful ABG interpretation and medication review.

## Introduction

Methaemoglobinaemia is a rare disorder caused by elevated levels of methaemoglobin in the blood. This forms when the iron in haem is oxidised from the ferrous state (Fe²⁺) to the ferric state (Fe³⁺). This, in turn, leads to higher oxygen affinity and reduced oxygen delivery to tissues. As the level rises, symptoms become apparent. These include cyanosis, lethargy, headache, and fatigue; at higher levels, it can also cause death. The rise in methaemoglobin levels can occur due to congenital or acquired causes. The majority of cases, however, are secondary to acquired causes, especially due to medications. The most common medication is dapsone, with other examples being chloroquine, benzocaine, and lidocaine [1]. Here, we discuss a subtle case of dapsone-induced methaemoglobinaemia presenting as subacute mental status changes and apparent hypoxia, which emphasises the importance of thorough medication reconciliation and maintaining a broad differential diagnosis.

## Case presentation

A 64-year-old patient with stage B chronic lymphocytic leukaemia presented for her routine chemotherapy (fludarabine + cyclophosphamide + rituximab). She was found to be hypoxic on pulse oximetry and was transferred to our centre with suspicion of pulmonary embolism, even though the patient was asymptomatic.

On further questioning, she was noted to be a non-smoker and to have occasional alcohol intake. She was recently diagnosed with CLL and had started chemotherapy three months prior to presentation. Prophylactic treatment against *Pneumocystis carinii *pneumonia (PCP) with dapsone had also been commenced.

**Figure 1:**
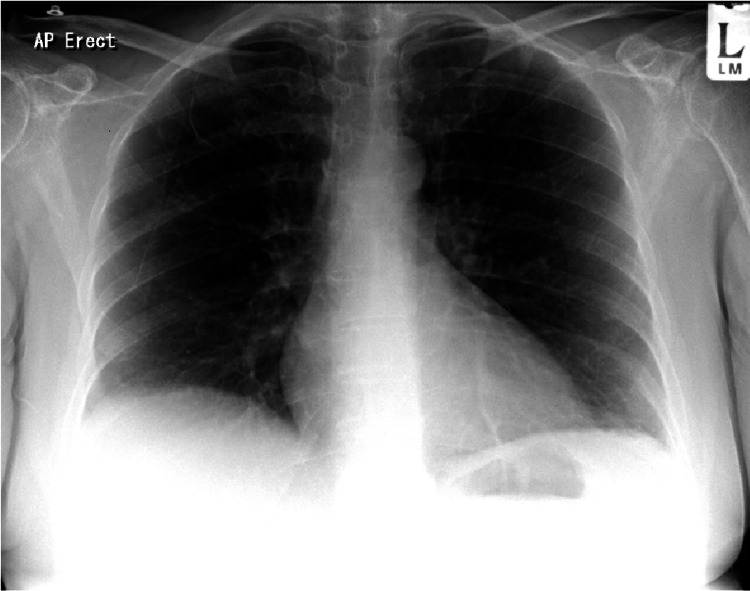
Initial normal chest X-ray

On examination, she appeared well, with normal vital signs. Clinically, she was not in distress apart from the apparent hypoxia visible on pulse oximetry. The National Early Warning Score (NEWS) was 5, based on SpO₂ 89% on 60% oxygen via a re-breathable face mask, pulse of 89 bpm (beats per minute), blood pressure of 153/82 mmHg, respiratory rate of 16, and temperature of 36.7°C. Chest examination was unremarkable, with bloods revealing mild leucopenia (3.0). The chest X-ray showed no significant acute abnormalities (Figure 1). Arterial blood gas analysis was performed to determine the level and cause of hypoxia, which revealed PaO₂ 65.6 kPa (on oxygen), PaCO₂ 5.0 kPa, HCO₃ 26.4 mmol/L, lactate 1.5 mmol/L, and methaemoglobin level 13.40% (normal 0-1.5%) (Table 1). Her Wells score was calculated due to suspicion of PE and was 1.

**Table 1:** Comparison of ABG analysis at different time points This table shows the comparison of ABG analysis performed at multiple time points, showing changes in the patient's blood levels of methaemoglobin. ABG: arterial blood gas

ABGs	Normal	Initial	Repeat Admission	After Treatment	Repeat After Treatment
pH	7.35 – 7.45	7.46	7.451	7.418	7.43
PaCo2	4.27 – 6.4 kPa	5 kPa	5.5 kPa	5.6 kPa	4.9 kPa
PaO2	11.04 – 14.36 kPa	65.6 kPa	11.2 kPa	14.2 kPa	16.2 kPa
HCO3	22 – 29 mmol/L	26.4 mmol/L	28.1 mmol/L	26.5 mmol/L	23.9 mmol/L
SpO2	94% – 98%	100%	96.70%	98.60%	98.40%
Oxyhaemoglobin levels	94% – 98%	86.30%	84.20%	91.30%	95.40%
Methaemoglobin levels	0% – 1.5%	13.40%	12.20%	7%	2.50%
Lactate levels	1.0 – 1.8 mmol/L	1.5 mmol/L	1.2 mmol/L	2.2 mmol/L	1.8 mmol/L

The ABG results were discussed with the on-call haematology consultant. The patient was moved to the haematology ward. The methaemoglobin levels were available on the gas analyser machine but were overlooked. This was due to a multitude of factors, including the infrequency with which this condition is encountered on acute medical wards, multiple comorbidities, and the appropriate clinical bias towards excluding more prevalent, dangerous, and common aetiologies.

**Figure 2:**
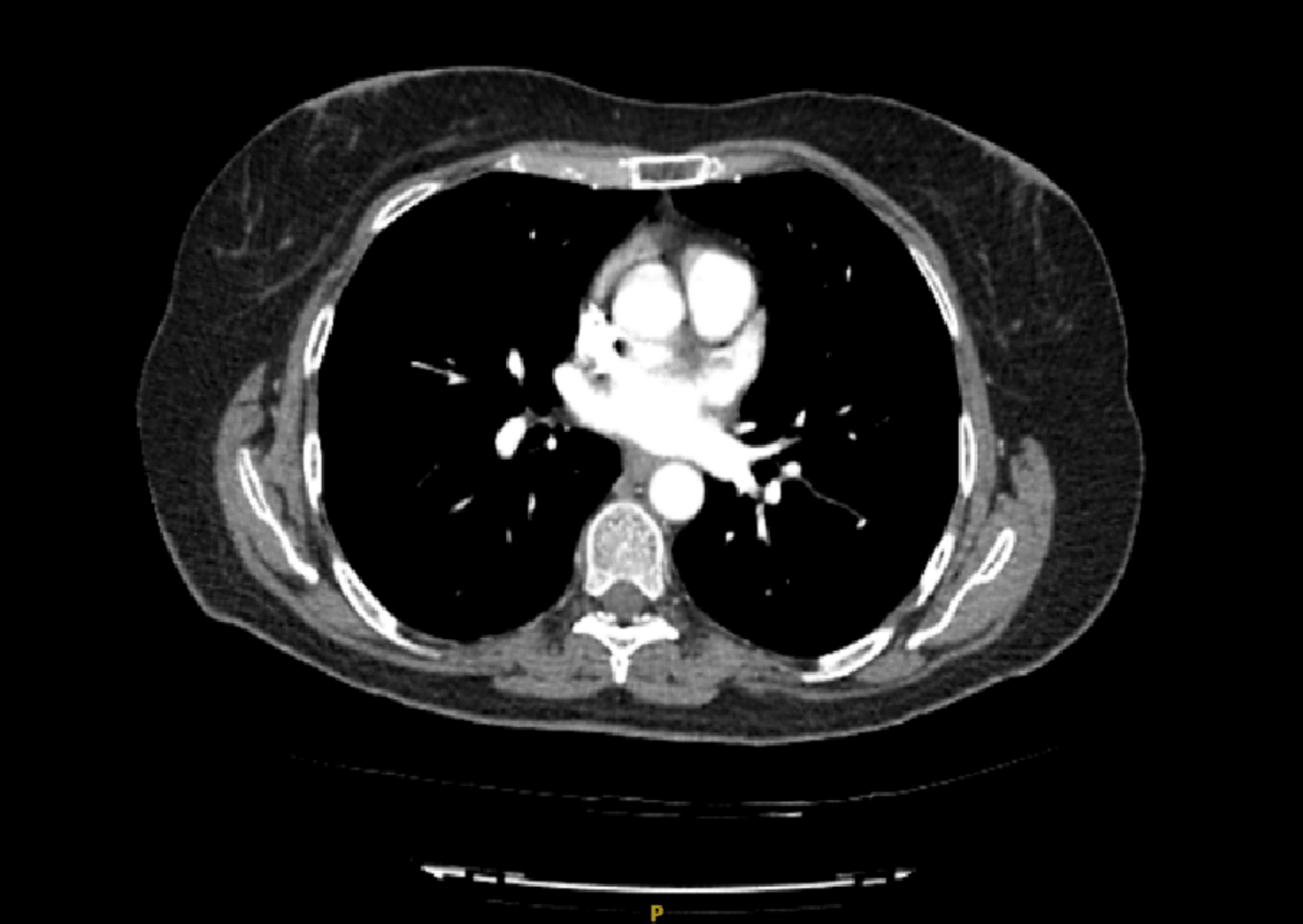
CTPA cross-sectional image The CTPA did not show any pulmonary emboli. CTPA: computed tomography pulmonary angiogram

The patient was extensively investigated for her hypoxia, including a computed tomography pulmonary angiogram (CTPA), which excluded pulmonary embolism (Figure 2). The patient was referred to the respiratory department for further investigations, including the possibility of pulmonary hypertension secondary to chemotherapy drugs. A 2D echocardiogram was arranged and was within normal limits. Spirometry excluded underlying obstructive or restrictive lung diseases. The patient was discharged with a prescription for dapsone, which is still ongoing, as well as a plan to follow up with a physician in due course. 

**Figure 3:**
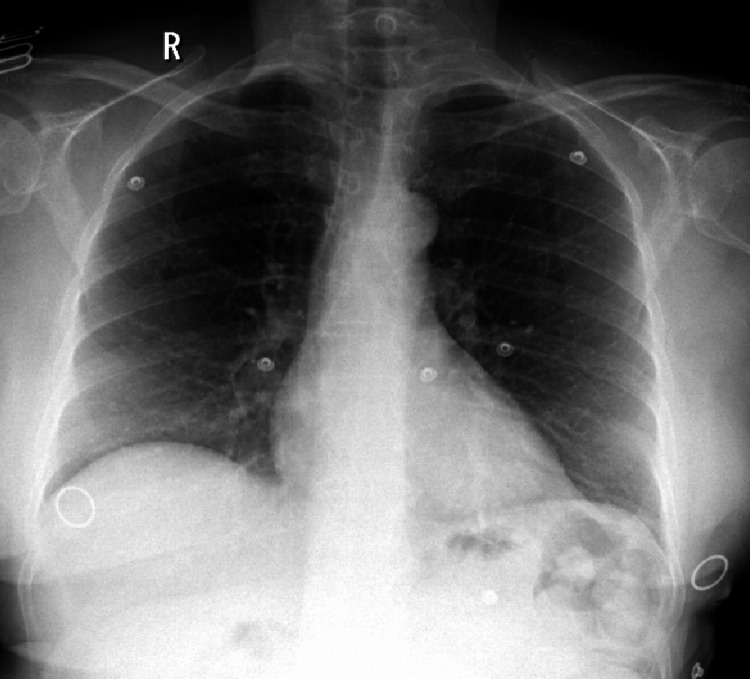
Chest X-ray on re-admission The repeat chest X-ray was noted to be normal.

The patient, however, presented again a month later with similar findings of hypoxia and no symptoms. Emergency workup, including a chest X-ray, did not show any changes (Figure 3). A second ABG showed methaemoglobin levels of 12.2% (normal 0-1.5%). This time, methaemoglobinaemia was identified, primarily because clinical bias was minimised given the patient’s previous admission, extensive investigations, and recurrence.

A diagnosis of methaemoglobinaemia was made, supported by the presence of a PaO₂ saturation gap. The patient was treated with methylene blue and vitamin C, after which the levels of methaemoglobin dropped - the first ABG showing levels of 7.0%, then 6.6%, and eventually 2.5%. Dapsone was discontinued, and the hypoxia resolved. The patient was later discharged. 

## Discussion

**Figure 4:**
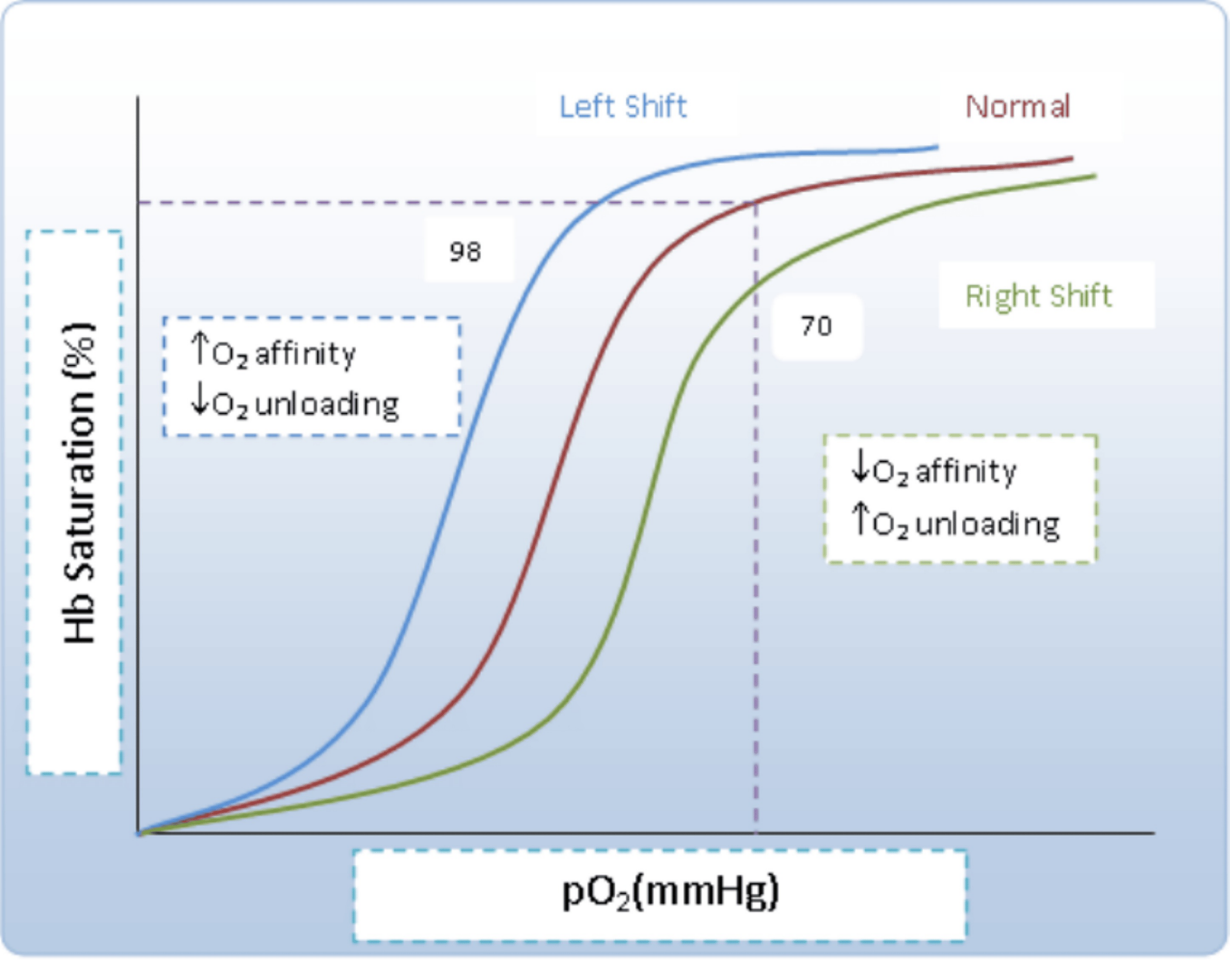
Oxygen dissociation curve This oxygen dissociation curve shows the affinity changes with Hb saturation and PO2 levels; at high Hb saturation affinity increases and unloading of O_2 _to tissue reduces. (Illustration by Dr. Harshvardhan Thakar)

The diagnosis of methaemoglobinaemia is suspected when the oxygen saturation is lower than 90% and does not improve after administration of oxygen; it is confirmed by high oxygen pressure in arterial blood gas [2]. The discrepancy between high partial pressure in arterial blood gas and low saturation levels can be further explored with the help of the oxygen dissociation curve. As Fe²⁺ converts to Fe³⁺, the oxygen dissociation curve shifts to the left, leading to reduced oxygen delivery to tissues (Figure 4). Hypoxia follows, which is apparent on pulse oximetry, but because haemoglobin is saturated with oxygen, PaO₂ will still be high on ABG sampling.

Methaemoglobinaemia can be congenital or acquired. The majority of cases are secondary to medications or exogenous agents [1]. Acquired methaemoglobinaemia occurs due to drugs with an oxidising effect, such as dapsone, sulphonamides, nitroglycerin, rasburicase, local anaesthetics, and more than two doses of methylene blue [2]. Dapsone (4,4′-diaminodiphenyl sulfone) is a sulfone antibiotic and potent anti-inflammatory that inhibits folate synthesis. Although it is traditionally an anti-leprosy drug, the use of dapsone has expanded into the treatment of dermatological disorders, rheumatological conditions, especially systemic lupus erythematosus, immune-deficiency-related infections such as *Pneumocystis jirovecii*, and even severe aphthous ulcers. Metabolism of this drug occurs via the cytochrome P450 pathway to potent oxidants responsible for adverse effects on red blood cells, namely haemolytic anaemia and methaemoglobinaemia [3]. Even though it is part of the treatment regimen for methaemoglobinaemia, methylene blue at higher doses can also precipitate methaemoglobinaemia.

## Conclusions

Hypoxia is always a red flag and requires prompt investigation and treatment. Although the cause of hypoxia is often apparent, there are times when it presents challenges, necessitating a thorough review of the patient’s medication history. Even though there are obvious life-threatening ailments that can cause hypoxia, requiring long, strenuous testing and multiple investigations, a simple test like ABG and its thorough analysis with minimisation of clinical bias could help differentiate one non-obvious and uncommon cause of hypoxia, such as methaemoglobinaemia.

## References

[1] Lewis JS, Jacobs ZG (2020). Subtle case of dapsone-induced methaemoglobinaemia. BMJ Case Rep.

[2] Islami MM (2024). Dapsone-induced methemoglobinemia case report. J Microsc Ultrastruct.

[3] Burke P, Jahangir K, Kolber MR (2013). Dapsone-induced methemoglobinemia: Case of the blue lady. Can Fam Physician.

